# Association between exposure to polycyclic aromatic hydrocarbons and endometriosis: data from the NHANES 2001–2006

**DOI:** 10.3389/fpubh.2023.1267124

**Published:** 2024-01-08

**Authors:** Li Zhang, Xiaojun Yang

**Affiliations:** Department of Obstetrics and Gynecology, The First Affiliated Hospital of Soochow University, Suzhou, Jiangsu, China

**Keywords:** polycyclic aromatic hydrocarbon, exposure, endometriosis, NHANES, BKMR

## Abstract

**Aim:**

To evaluate the association between urinary polycyclic aromatic hydrocarbon (PAH) metabolites and the risk of endometriosis.

**Methods:**

This cross-sectional study obtained data on women aged 20–54 years from the National Health and Nutrition Examination Survey (NHANES) 2001–2006. The weighted multivariate logistic regression model was established to assess the association between the eight urinary PAH metabolites and the risk of endometriosis. In this multivariate analysis, the eight urinary PAH metabolites were adjusted with urinary creatinine, and were divided into three groups according to tertiles: Tertile 1, Tertile 2 and Tertile 3. To evaluate the overall association of mixed PAH metabolites with endometriosis, the Bayesian kernel machine regression (BKMR) model was applied.

**Results:**

Totally 1,291 women were included, of which 90 (6.97%) had endometriosis and 1,201 (93.03%) did not have endometriosis. After adjusting for age, race, smoking, age at menarche, hysterectomy, ovary removed, female hormone use, and menopause, compared with the Tertile 1 group, the Tertile 2 and Tertile 3 groups of all PAH metabolites demonstrated no significant risk of endometriosis. A positive tendency was found between mixed PAH metabolites and endometriosis when all the metabolites were at their 60th percentile levels or above compared with their median levels. When all the other metabolites were fixed at their median levels, 1-hydroxynaphthalene was positively correlated with endometriosis. Potential interactions existed between 1-hydroxynaphthalene and 2-hydroxynaphthalene and between 2-hydroxyfluorene and 3-hydroxyfluorene.

**Conclusion:**

No significant association was found between individual PAH metabolites and endometriosis. A positive association existed between mixed PAH metabolites and the risk of endometriosis.

## Introduction

Endometriosis, defined as the presence of endometrial tissue outside the uterus, is a chronic gynecological disease with an increased incidence, affecting 5–10% of women of childbearing age and causing pelvic pain and infertility (1–3). Painful periods, deep dyspareunia, dyschezia, dysuria, and abdominal pain are the most common symptoms (4, 5). Endometriosis is associated with a 50% increase in the risk of epithelial ovarian cancer (6), and is also correlated with gastrointestinal and immunological diseases and thyroid cancer (7). Endometriosis is an estrogen-dependent disease, which can be affected by genetics, environmental factors, and the immune system (8, 9).

Environmental endocrine-disrupting chemicals (EDCs) are a kind of exogenous chemicals that interfere with the endocrine system, which can affect the endocrine system and reproductive health (10, 11). Exposure to EDCs has been suggested to be associated with the increasing incidence of endometriosis (12, 13). Evidence shows that EDCs such as phthalates, perfluoroalkyl substances and heavy metals are related to endometriosis (14–16). Polycyclic aromatic hydrocarbons (PAHs), as a kind of EDC, comprise two or more condense aromatic rings and have a toxic nature, threatening to public health (17). PAHs mainly exist in polluted air, soil, water and food (18, 19). PAHs can enter the human body through different occupational, environmental, medical, and nutritional sources, such as exposure to aluminum and graphite electrode production or road construction, dietary intake of meat, dairy products or smoked foods, etc. (20, 21). Humans are exposed to PAHs through inhalation, ingestion and skin contact (22). PAH metabolites in human urine can be utilized as internal dosage indicators to measure PAH exposure (23). At present, the associations between PAH exposure and sex hormone levels (e.g., reproductive hormones, estradiol, and serum testosterone) have been reported (24–27), whereas no study has assessed the association between PAH exposure and the risk of endometriosis. Of note, individuals are exposed to various chemicals at the same time, resulting in a mixture exposure pattern. It is necessary to explore the individual and combined association between urinary PAH metabolites and the risk of endometriosis, so as to promote understanding of the relationship between PAH exposure and endometriosis, and provide reference for the prevention and management of endometriosis.

The objective of this study was to evaluate the association of urinary individual and mixed PAH metabolites with the risk of endometriosis, using the data from the National Health and Nutrition Examination Survey (NHANES) 2001–2006.

## Methods

### Study design and population

This cross-sectional study obtained data from the NHANES 2001–2006 (2001–2002, 2003–2004, 2005–2006). Since information on endometriosis assessment was only available in the NHANES 1999–2006, and data on urinary PAH metabolites (1-hydroxynaphthalene, 2-hydroxynaphthalene, 2-hydroxyfluorene, 3-hydroxyfluorene, 1-hydroxyphenanthrene, 2-hydroxyphenanthrene, 3-hydroxyphenanthrene, 1-hydroxypyrene) were only available in the NHANES 2001–2006, this study chose the timeline 2001–2006. The NHANES is a cross-sectional survey that collects demographic, clinical, behavioral, dietary, social, and laboratory data on the health and nutritional status of the non-institutionalized population in the United States, employing a multilayered probability sampling design (28). The survey was approved by the Institutional Review Board of the National Center for Health Statistics (NCHS), and all participants provided informed consent. Since the data of the NHANES were de-identified, further approval by the institutional review board was waived. Women aged 20–54 years were included in this study since endometriosis was only evaluated in women aged 20–54 years in the NHANES. Participants (1) without the assessment of endometriosis and (2) not examined for urinary PAH concentrations in the NHANES were excluded.

### Urinary PAH metabolite measurement

Participants aged 6 years and older were eligible for the measurement of urinary PAH metabolites, including 1-hydroxynaphthalene, 2-hydroxynaphthalene, 2-hydroxyfluorene, 3-hydroxyfluorene, 1-hydroxyphenanthrene, 2-hydroxyphenanthrene, 3-hydroxyphenanthrene, 1-hydroxypyrene, using capillary gas chromatog-raphy combined with high resolution mass spectrometry (GC–HRMS). Urine samples were collected by experienced technicians and were stored at −20°C. Details of the measurement are illustrated on the NHANES website (29). Values below the lower limit of detection (LLOD) were calculated as LLOD/√2 according to the NHANES laboratory. Considering the potential bias caused by kidney condition, the urinary PAH metabolites (ng/L) were adjusted/divided by urinary creatinine (mg/dL).

### Endometriosis assessment

Whether participants had endometriosis was assessed according to the question “Has a doctor or other health professional ever told {you/SP} that {you/she} had endometriosis?” If the response was “yes,” the participant was regarded to have endometriosis.

### Covariates

The collected covariates included age (years), race, education level, marital status, poverty-to-income ratio (PIR), drinking, smoking, body mass index (BMI, kg/m^2^), circumference (cm), creatinine (mg/dL), cotinine (ng/mL), age at menarche (years), hysterectomy, gravidity, ovary removed, female hormone use, pregnancy times, and menopause. Race was classified into Mexican American, non-Hispanic black, non-Hispanic white, other Hispanic, and other race. Education level was divided into college graduate or above, some college or associate (AA) degree, high school graduate/general education development (GED), and less than high school. Marital status included married and other status. Participants were classified as underweight (<18.5 kg/m^2^), normal-weight (18.5 kg/m^2^–24.9 kg/m^2^), overweight (25.0 kg/m^2^–29.9 kg/m^2^), and obese (≥30 kg/m^2^) based on BMI (30). Study questions for covariate assessment are shown in Supplementary Table S1.

### Statistical analysis

Measurement data with normal distribution were described by mean ± standard deviation (Mean ± SD), and the independent-samples *t* test was used for comparisons between two groups. Measurement data with skewed distribution were shown as median and quartile [M (Q_1_, Q_3_)], and between-group comparisons were subject to the Wilcoxon rank sum test. Enumeration data were expressed as the number of cases and the composition ratio [n (%)], and the Chi-square test or Fisher’s exact test was applied for comparisons between groups. The missing data were interpolated by the random forest imputation method. The sensitivity analysis was carried out by comparing the data before and after the imputation to exclude the influence of the random forest imputation method on the results (Supplementary Table S2).

The participants were divided into endometriosis and non-endometriosis groups. After the difference analysis, the variables with significant differences between the two groups were taken as confounding factors. The Pearson correlation was employed to calculate the correlation coefficients among these PAH metabolites. Then the weighted multivariate logistic regression model was established to assess the association between the eight urinary PAH metabolites and the risk of endometriosis, and the confounding factors, age, race, smoking, age at menarche, hysterectomy, ovary removed, female hormone use, and menopause, were adjusted for in the model. Odds ratios (ORs) and 95% confidence intervals (CIs) were calculated. In this multivariate analysis, the eight urinary PAH metabolites were adjusted with urinary creatinine, and were divided into three groups according to tertiles: Tertile 1, Tertile 2, and Tertile 3, and then the study population was grouped based on the three groups of the urinary PAH metabolites. To evaluate the overall association of mixed PAH metabolites with endometriosis, the Bayesian kernel machine regression (BKMR) model was applied with a hierarchical variable selection method with 10,000 iterations. BKMR model assumptions include linear relationship assumption, Gaussian noise assumption, additive assumption, and prior distribution assumption. One of the BKMR model’s limitations is the kernel algorithm. Fixing other chemicals at certain levels in order to extrapolate the exposure-response function does not allow for estimation of the effects of co-exposure patterns with both high and low levels of chemicals. The effects of co-exposure patterns with both high and low levels of chemicals cannot be estimated when other chemicals are fixed at certain amounts to extrapolate the exposure-response function. Besides, the total risk is evaluated using the BKMR model with all of the chemicals at different percentiles relative to their median exposure levels, whereas the actual exposure pattern is a mixture of chemicals with varying levels of exposure. 2-hydroxyfluorene and 3-hydroxyfluorene had a strong correlation (*r* = 0.87), and were grouped into the first group (group 1); 1-hydroxyphenanthrene, 2-hydroxyphenanthrene, 3-hydroxyphenanthrene had similar exposure sources, and 1-hydroxyphenanthrene and 2-hydroxyphenanthrene had a strong correlation (*r* = 0.81), so these three metabolites were grouped into the second group (group 2); the remaining 1-hydroxynaphthalene, 2-hydroxynaphthalene and 1-hydroxypyrene had weak correlations with each other, and were grouped into the third group (group 3). The joint effect was calculated by comparing all the PAH metabolites at their 60th percentile levels or above with all of them at their 50th percentile. The group posterior inclusion probability (groupPIP) and conditional PIP (condPIP) indicated the probability of the group and the PAH metabolite in each group included in the model and represented their contribution to the overall association. By fixing all the other PAH metabolites at their median levels, the exposure-response relationship was explored in the BKMR model. BKMR bivariable analysis was conducted to explore the relationship between individual PAH metabolites and endometriosis, while fixing another PAH metabolite at the 10th, 50th, and 90th quantiles (and keeping the remaining PAH metabolites at their median levels), so as to assess the interactions between any two of the PAH metabolites. Prior studies showed that when variables used to calculate sampling weights were included in the analysis, the weighted estimation could cause over-adjusted bias (31, 32). In addition, it was unclear whether sampling weights were suitable for BKMR, a complex statistical model. Thus, sampling weights were not applied in the BKMR model.

Data cleaning (including missing value statistics) and missing value imputation were completed using Python 3.7.4 (Python Software Foundation, Delaware, United States). Sensitivity analysis and difference comparison were completed by SAS 9.4 (SAS Institute Inc., Cary, NC, United States). The weighted multivariate logistic modeling and BKMR modeling were completed by R 4.2.1 (Institute for Statistics and Mathematics, Vienna, Austria). *p* < 0.05 indicated statistically significant.

## Results

### Participant characteristics

**Figure 1:**
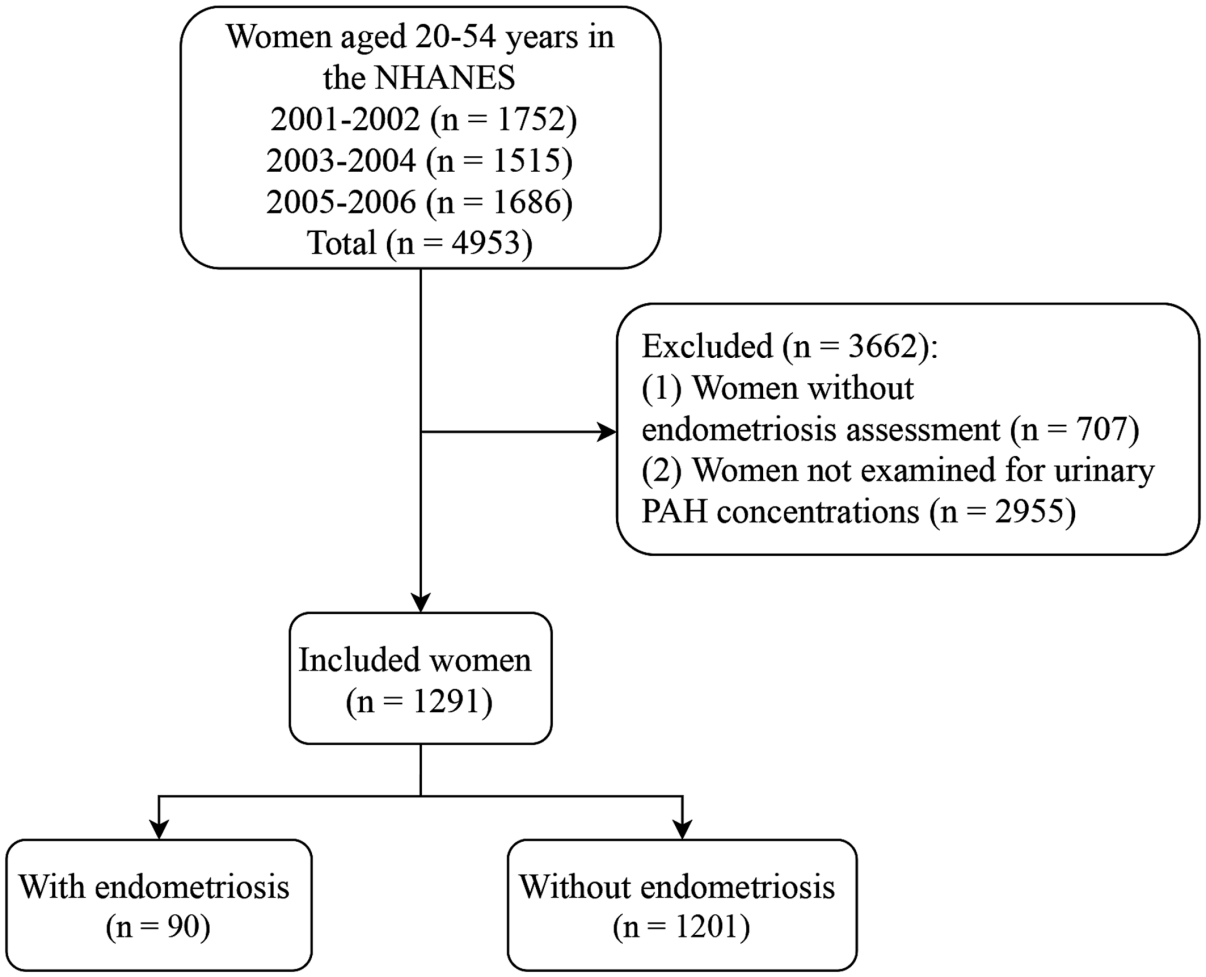
Flow chart of participant selection. NHANES, the National Health and Nutrition Examination Survey; PAH, polycyclic aromatic hydrocarbon.

After excluding participants without the assessment of endometriosis (*n* = 707) and not examined for urinary PAH concentrations (*n* = 2,955), 1,291 women were included in this study, of which 90 (6.97%) had endometriosis and 1,201 (93.03%) did not have endometriosis. Figure 1 presents the flow chart of participant selection. The average age of the included women was 35.66 years. There were significant differences between the endometriosis and non-endometriosis groups in age, race, smoking, age at menarche, hysterectomy, ovary removed, female hormone use, and menopause (all *p* < 0.05). The characteristics of the included women are shown in Table 1. The endometriosis group had significantly more 1-hydroxynaphthalene, 2-hydroxyfluorene, 3-hydroxyfluorene, 1-hydroxyphenanthrene, 2-hydroxyphenanthrene, and 3-hydroxyphenanthrene than the non-endometriosis group (all *p* < 0.05).

**Table 1:** Characteristics of the included women.

Variables	Total (*n* = 1,291)	Non-endometriosis group (*n* = 1,201)	Endometriosis group (*n* = 90)	Statistics	*p*
Age, years, Mean ± SD	35.66 ± 10.13	35.35 ± 10.12	39.80 ± 9.31	*t* = −4.04	<0.001
Race, *n* (%)				χ^2^ = 13.916	0.008
Mexican American	283 (21.92)	275 (22.90)	8 (8.89)		
Non-Hispanic black	272 (21.07)	255 (21.23)	17 (18.89)		
Non-Hispanic white	624 (48.33)	567 (47.21)	57 (63.33)		
Other Hispanic	45 (3.49)	42 (3.50)	3 (3.33)		
Other race	67 (5.19)	62 (5.16)	5 (5.56)		
Education, *n* (%)				χ^2^ = 2.822	0.420
College graduate or above	291 (22.54)	271 (22.56)	20 (22.22)		
Some college or AA degree	428 (33.15)	396 (32.97)	32 (35.56)		
High school graduate /GED	288 (22.31)	264 (21.98)	24 (26.67)		
Less than high school	284 (22.00)	270 (22.48)	14 (15.56)		
Marital status *n* (%)				χ^2^ = 1.169	0.280
Married	733 (56.78)	677 (56.37)	56 (62.22)		
Other	558 (43.22)	524 (43.63)	34 (37.78)		
PIR, M (Q_1_, Q_3_)	2.49 (1.21, 4.42)	2.47 (1.20, 4.39)	3.16 (1.34, 4.74)	*Z* = 1.488	0.137
Drinking, *n* (%)				χ^2^ = 0.472	0.492
No	503 (38.96)	471 (39.22)	32 (35.56)		
Yes	788 (61.04)	730 (60.78)	58 (64.44)		
Smoking, *n* (%)				χ^2^ = 8.073	0.004
No	798 (61.81)	755 (62.86)	43 (47.78)		
Yes	493 (38.19)	446 (37.14)	47 (52.22)		
BMI, kg/m^2^, Mean ± SD	28.73 ± 7.26	28.75 ± 7.29	28.47 ± 6.83	*t* = 0.35	0.726
BMI, *n* (%)				χ^2^ = 2.613	0.455
Underweight	34 (2.63)	33 (2.75)	1 (1.11)		
Normal	392 (30.36)	363 (30.22)	29 (32.22)		
Overweight	397 (30.75)	365 (30.39)	32 (35.56)		
Obesity	468 (36.25)	440 (36.64)	28 (31.11)		
Circumference, cm, Mean ± SD	94.76 ± 16.15	94.73 ± 16.22	95.10 ± 15.25	*t* = −0.21	0.835
Creatinine, mg/dL, M (Q_1_, Q_3_)	113.00 (64.00, 164.00)	113.00 (64.00, 162.00)	113.00 (61.00, 173.00)	*Z* = 0.357	0.721
Cotinine, ng/mL, M (Q_1_, Q_3_)	0.07 (0.02, 4.29)	0.07 (0.02, 3.19)	0.09 (0.02, 126.00)	*Z* = 1.001	0.317
Age at menarche, years, Mean ± SD	12.61 ± 1.78	12.64 ± 1.77	12.25 ± 1.85	*t* = 2.00	0.045
Hysterectomy, *n* (%)				χ^2^ = 75.320	<0.001
No	1,180 (91.40)	1,120 (93.26)	60 (66.67)		
Yes	111 (8.60)	81 (6.74)	30 (33.33)		
Gravidity, *n* (%)				χ^2^ = 2.220	0.136
No	1,058 (81.95)	979 (81.52)	79 (87.78)		
Yes	233 (18.05)	222 (18.48)	11 (12.22)		
Ovary removed, *n* (%)				χ^2^ = 96.503	<0.001
No	1,191 (92.25)	1,132 (94.25)	59 (65.56)		
Yes	100 (7.75)	69 (5.75)	31 (34.44)		
Female hormones use, *n* (%)				χ^2^ = 36.592	<0.001
No	994 (76.99)	948 (78.93)	46 (51.11)		
Yes	297 (23.01)	253 (21.07)	44 (48.89)		
Pregnancy times, *M* (Q_1_, Q_3_)	2.89 (2.00, 4.00)	2.83 (2.00, 4.00)	3.00 (2.00, 4.00)	*Z* = 0.594	0.553
Menopause, *n* (%)				χ^2^ = 37.535	<0.001
No	1,084 (83.97)	1,029 (85.68)	55 (61.11)		
Yes	207 (16.03)	172 (14.32)	35 (38.89)		
1-hydroxynaphthalene, ng/L, M (Q_1_, Q_3_)	1741.00 (762.60, 5981.00)	1683.40 (758.00, 5483.00)	3325.75 (1095.00, 12167.00)	*Z* = 2.894	0.004
2-hydroxynaphthalene, ng/L, M (Q_1_, Q_3_)	3434.50 (1525.50, 8717.00)	3317.10 (1514.00, 8548.70)	4787.85 (1911.00, 13118.90)	*Z* = 1.897	0.058
2-hydroxyfluorene, ng/L, M (Q_1_, Q_3_)	257.00 (129.00, 594.90)	247.00 (126.30, 578.20)	345.65 (193.10, 1109.50)	*Z* = 2.706	0.007
3-hydroxyfluorene, ng/L, M (Q_1_, Q_3_)	87.00 (42.10, 247.00)	85.00 (42.10, 232.00)	134.50 (53.00, 609.00)	*Z* = 2.443	0.015
1-hydroxyphenanthrene, ng/L, M (Q_1_, Q_3_)	152.00 (82.00, 291.70)	150.50 (82.00, 284.90)	188.15 (93.10, 395.00)	*Z* = 2.017	0.044
2-hydroxyphenanthrene, ng/L, M (Q_1_, Q_3_)	60.00 (30.20, 126.70)	59.60 (30.00, 124.70)	74.10 (39.00, 165.50)	*Z* = 2.145	0.032
3-hydroxyphenanthrene, ng/L, M (Q_1_, Q_3_)	89.70 (46.00, 174.00)	88.00 (45.90, 172.50)	104.45 (56.00, 213.40)	*Z* = 2.119	0.034
1-hydroxypyrene, ng/L, M (Q_1_, Q_3_)	72.50 (34.00, 157.00)	71.40 (34.00, 157.00)	78.15 (40.00, 169.80)	*Z* = 1.141	0.254

PIR, poverty-to-income ratio; BMI, body mass index; AA, associate; GED, general education development; SD, standard deviation; M, median; Q_1_, 1st quartile; Q_3_, 3rd quartile.

### Distribution and correlation of urinary PAH metabolites

**Figure 2:**
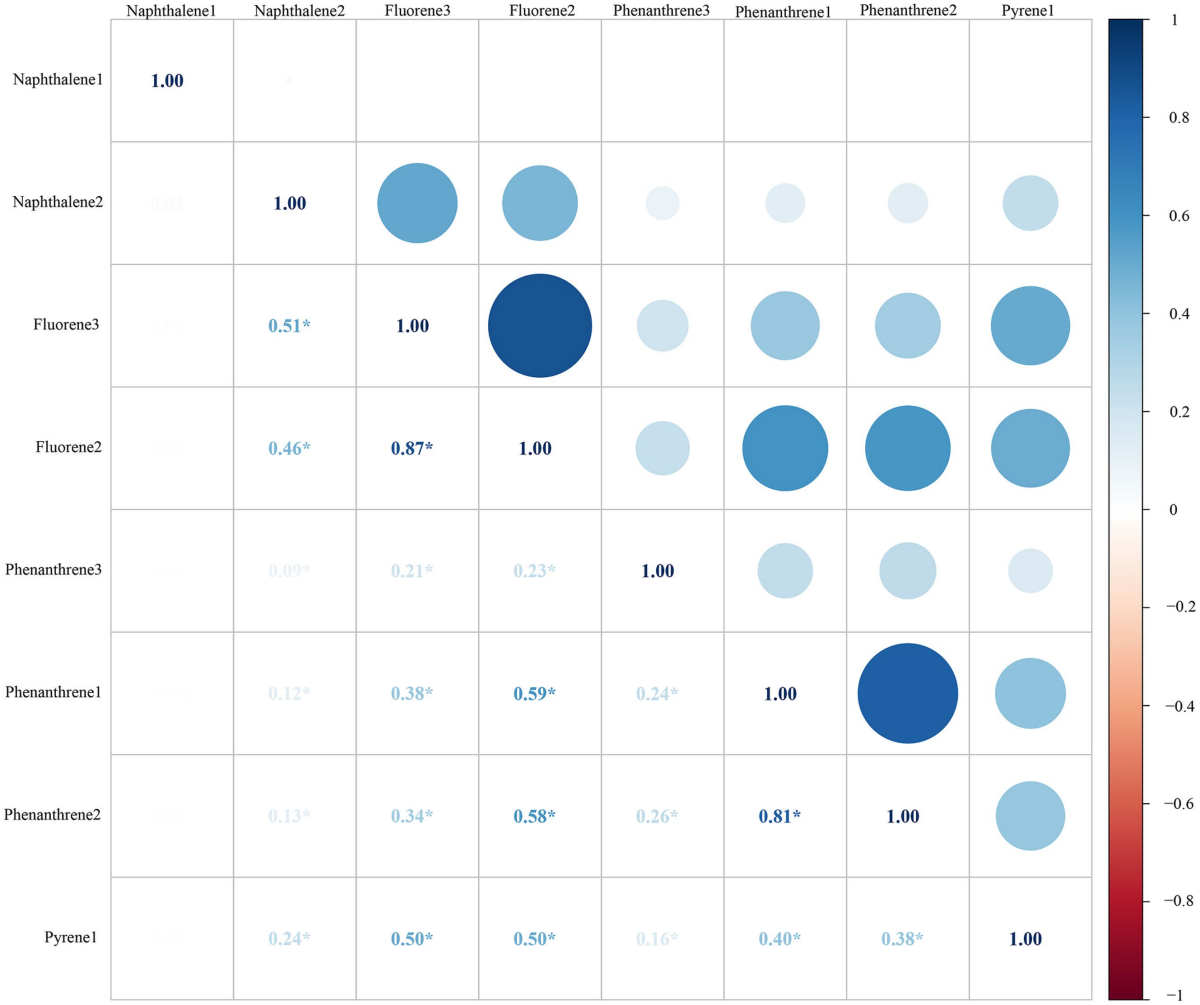
Pearson correlations between urinary PAH metabolites. All the correlations were statistically significant (all *p* < 0.05), except those of 1-NAP and any other metabolites. All the urinary PAH metabolites (ng/L) were adjusted/divided by urinary creatinine (mg/dL).*: *p* > 0.05. PAH, polycyclic aromatic hydrocarbon; Naphthalene1, 1-hydroxynaphthalene; Naphthalene2, 2-hydroxynaphthalene; Fluorene2, 2-hydroxyfluorene; Fluorene3, 3-hydroxyfluorene; Phenanthrene1, 1-hydroxyphenanthrene; Phenanthrene2, 2-hydroxyphenanthrene; Phenanthrene3, 3-hydroxyphenanthrene; Pyrene1, 1-hydroxypyrene.

Table 2 shows the geometric mean (GM) values and intervals (Tertile 1, Tertile 2 and Tertile 3) of the eight urinary PAH metabolites, and 2-hydroxynaphthalene (GM = 35.875, 95%CI: 32.756–38.993) and 2-hydroxyphenanthrene (GM = 0.599, 95%CI: 0.550, 0.648) had the highest and lowest GM values, respectively. No significant correlation was found between 1-hydroxynaphthalene and any other metabolites. There were significant positive correlations between all other metabolites (all *p* < 0.05) (Figure 2).

**Table 2:** Distribution of urinary PAH metabolites.

Variables	GM (95%CI)	Tertile 1	Tertile 2	Tertile 3
1-hydroxynaphthalene	24.898 (22.589, 27.207)	<10.055	10.055 – 35.627	≥35.627
2-hydroxynaphthalene	35.875 (32.756, 38.993)	<20.708	20.708 – 54.384	≥54.384
2-hydroxyfluorene	3.217 (2.953, 3.481)	<1.643	1.643 – 3.536	≥3.536
3-hydroxyfluorene	1.317 (1.200, 1.434)	<0.557	0.557 – 1.385	≥1.385
1-hydroxyphenanthrene	1.564 (1.464, 1.665)	<1.082	1.082 – 1.968	≥1.968
2-hydroxyphenanthrene	0.599 (0.550, 0.648)	<0.436	0.436 – 0.824	≥0.824
3-hydroxyphenanthrene	1.008 (0.943, 1.072)	<0.613	0.613 – 1.168	≥1.168
1-hydroxypyrene	0.744 (0.688, 0.799)	<0.484	0.484 – 1.032	≥1.032

All the urinary PAH metabolites (ng/L) were adjusted/divided by urinary creatinine (mg/dL). PAH, polycyclic aromatic hydrocarbon; GM, geometric mean; CI, confidence interval.

### Association between individual PAH metabolites and endometriosis

**Figure 3:**
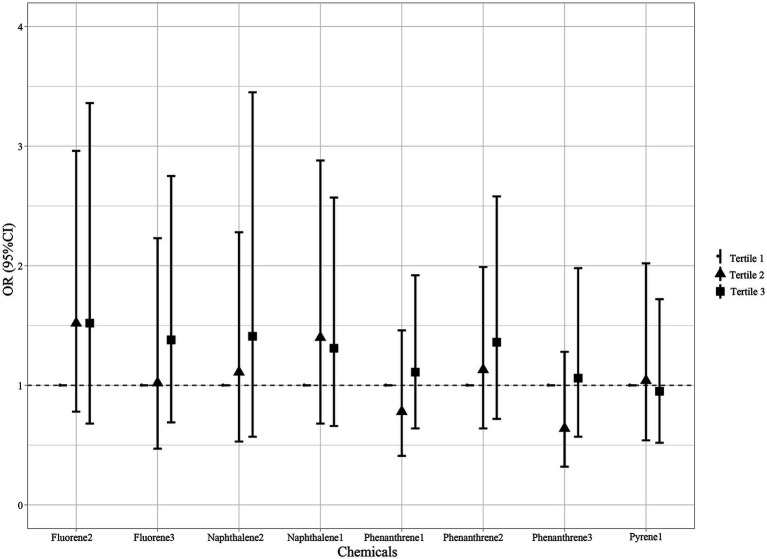
Association between individual PAH metabolites and endometriosis. Models were adjusted for age, race, smoking, age at menarche, hysterectomy, ovary removed, female hormone use, and menopause. All the urinary PAH metabolites (ng/L) were adjusted/divided by urinary creatinine (mg/dL). PAH, polycyclic aromatic hydrocarbon; Naphthalene1, 1-hydroxynaphthalene; Naphthalene2, 2-hydroxynaphthalene; Fluorene2, 2-hydroxyfluorene; Fluorene3, 3-hydroxyfluorene; Phenanthrene1, 1-hydroxyphenanthrene; Phenanthrene2, 2-hydroxyphenanthrene; Phenanthrene3, 3-hydroxyphenanthrene; Pyrene1, 1-hydroxypyrene; OR, odds ratios; CI, confidence interval.

After adjusting for age, race, smoking, age at menarche, hysterectomy, ovary removed, female hormone use, and menopause, compared with the Tertile 1 group, the Tertile 2 and Tertile 3 groups of all PAH metabolites demonstrated no significant risk of endometriosis (Figure 3).

### Overall association between mixed PAH metabolites and endometriosis

**Figure 4:**
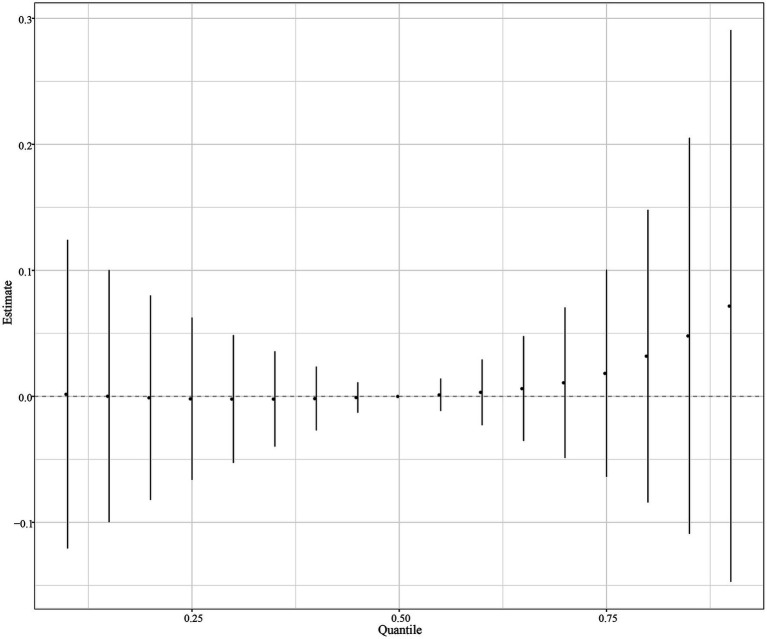
Overall association between mixed PAH metabolites and endometriosis when all the metabolites were at their 60th percentile levels or above compared with their median levels. Models were adjusted for age, race, smoking, age at menarche, hysterectomy, ovary removed, female hormone use, and menopause. All the urinary PAH metabolites (ng/L) were adjusted/divided by urinary creatinine (mg/dL), and underwent log conversion. PAH, polycyclic aromatic hydrocarbon.

**Figure 5:**
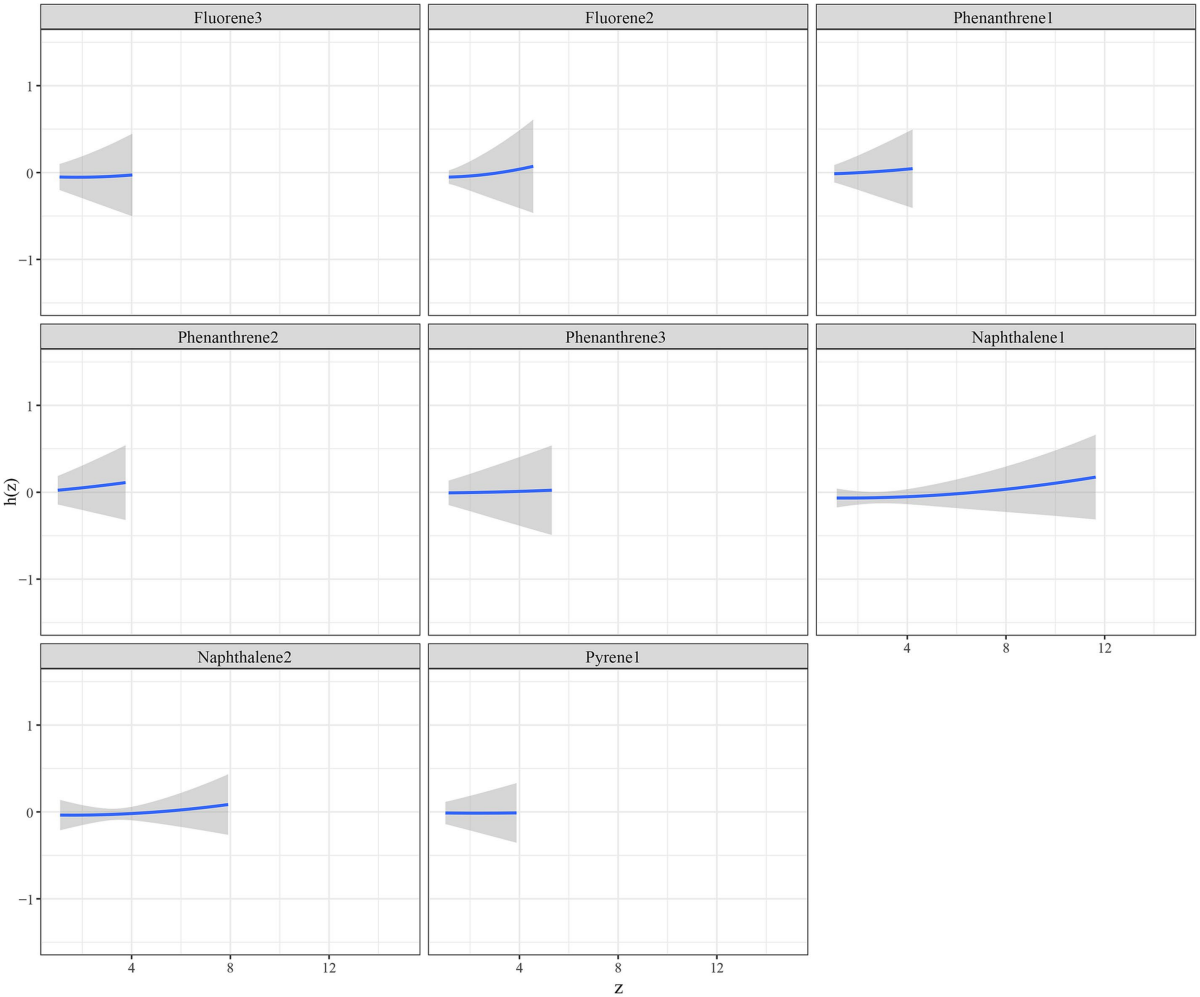
Trends of exposure-response functions of PAH metabolites when all the other metabolites were fixed at their median levels. Models were adjusted for age, race, smoking, age at menarche, hysterectomy, ovary removed, female hormone use, and menopause. All the urinary PAH metabolites (ng/L) were adjusted/divided by urinary creatinine (mg/dL), and underwent log conversion. PAH, polycyclic aromatic hydrocarbon; Naphthalene1, 1-hydroxynaphthalene; Naphthalene2, 2-hydroxynaphthalene; Fluorene2, 2-hydroxyfluorene; Fluorene3, 3-hydroxyfluorene; Phenanthrene1, 1-hydroxyphenanthrene; Phenanthrene2, 2-hydroxyphenanthrene; Phenanthrene3, 3-hydroxyphenanthrene; Pyrene1, 1-hydroxypyrene.

**Figure 6:**
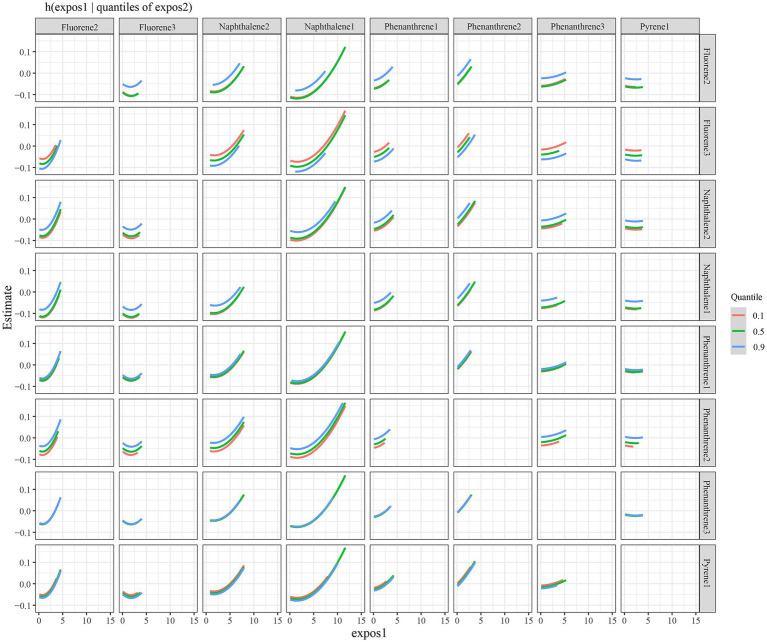
Interaction between urinary PAH metabolites, while fixing one of two PAH metabolites at the 10th, 50th, and 90th quantiles (and keeping the remaining PAH metabolites at their median levels). Models were adjusted for age, race, smoking, age at menarche, hysterectomy, ovary removed, female hormone use, and menopause. All the urinary PAH metabolites (ng/L) were adjusted/divided by urinary creatinine (mg/dL), and underwent log conversion. PAH, polycyclic aromatic hydrocarbon; Naphthalene1, 1-hydroxynaphthalene; Naphthalene2, 2-hydroxynaphthalene; Fluorene2, 2-hydroxyfluorene; Fluorene3, 3-hydroxyfluorene; Phenanthrene1, 1-hydroxyphenanthrene; Phenanthrene2, 2-hydroxyphenanthrene; Phenanthrene3, 3-hydroxyphenanthrene; Pyrene1, 1-hydroxypyrene.

The eight urinary PAH metabolites were grouped into three groups according to their similar exposure sources and correlations. After adjusting for the parameters, a positive tendency was found between mixed PAH metabolites and endometriosis when all the metabolites were at their 60th percentile levels or above compared with their median levels (Figure 4). Table 3 exhibits the contribution of all the PAH metabolites to the overall association. The group 1 had the highest groupPIP (groupPIP = 0.25), and 2-hydroxyfluorene made the most contribution in the group 1 (CondPIP = 0.64). The trends of exposure-response functions of the eight metabolites are illustrated in Figure 5. When all the other metabolites were fixed at their median levels, 1-hydroxynaphthalene was positively correlated with endometriosis. According to BKMR bivariable analysis, potential interactions existed between 1-hydroxynaphthalene and 2-hydroxynaphthalene and between 2-hydroxyfluorene and 3-hydroxyfluorene (Figure 6).

**Table 3:** Contribution of urinary PAH metabolites to the overall association.

Variables	Group	GroupPIP	CondPIP
2-hydroxyfluorene	1	0.25	0.64
3-hydroxyfluorene	1	0.25	0.36
1-hydroxyphenanthrene	2	0.13	0.36
2-hydroxyphenanthrene	2	0.13	0.18
3-hydroxyphenanthrene	2	0.13	0.46
1-hydroxynaphthalene	3	0.13	0.33
2-hydroxynaphthalene	3	0.13	0.50
1-hydroxypyrene	3	0.13	0.17

All the urinary PAH metabolites (ng/L) were adjusted/divided by urinary creatinine (mg/dL), and underwent log conversion. GroupPIP, group posterior inclusion probability; CondPIP, conditional posterior inclusion probability.

## Discussion

To our knowledge, this study was the first to evaluate the association between urinary PAH metabolites and endometriosis. The BKMR model was applied herein to estimate the overall association of mixed PAH metabolites with endometriosis. No significant association was found between individual PAH metabolites and the risk of endometriosis. There was a positive tendency between mixed PAH metabolites and endometriosis when all the metabolites were at their 60th percentile levels or above compared with their median levels, and when all the other metabolites were fixed at their median levels, 1-hydroxynaphthalene was positively correlated with endometriosis.

Increasing evidence showed an association between PAH metabolites and reproductive diseases, such as infertility and urogenital tract abnormalities (33, 34). Several studies has connected endometriosis with organic pollutants (12, 35, 36). For the relationship between PAH metabolites and endometriosis, individual PAH metabolites were reported to be not associated with the risk of endometriosis, according to multivariate logistic regression analysis in this paper. Since chemicals are exposed at the same time, and usually exposure sources and metabolism pathways can lead to high collinearity, the association between a chemical and health outcomes may be masked or exaggerated by other relevant chemicals (37, 38). Thus, chemical mixtures should be considered in assessing the association between PAH metabolites and endometriosis.

The BKMR model was applied to evaluate the overall association between mixed PAH metabolites and endometriosis. The current study found a positive trend between mixed PAH metabolites and endometriosis when all the metabolites were at their 60th percentile levels or above versus their median levels. Exposure to PAHs could affect female reproduction, and luteinizing hormone, follicle stimulating hormone, gonadotrophin releasing hormone, and aromatase seem to be influenced by PAHs (39). As endometriosis is estrogen-dependent (40), PAH exposure might be associated with an increased risk of endometriosis through adversely impacting the above hormonal regulators. We also demonstrated that when all the other metabolites were fixed at their median levels, 1-hydroxynaphthalene was positively correlated with endometriosis, which needs future evidence for validation due to the lack of studies. Notably, in the BKMR model, the overall association was assessed with all the PAH metabolites at their 60th percentile levels or above versus their median levels, but the exposure pattern in a real-world setting is a combination of PAH metabolites with different exposure levels. Thus, the results from BKMR analysis should be interpreted cautiously. In addition, there may be interactions between 1-hydroxynaphthalene and 2-hydroxynaphthalene and between 2-hydroxyfluorene and 3-hydroxyfluorene on the risk of endometriosis. As a possible implication, exposure to PAHs should be reduced to prevent or lower the risk of endometriosis, for example, via raising public awareness of PAHs and environmental measures.

The NHANES uses a complex, multistage, probability sampling design, and assesses a nationally representative sample of around 5,000 persons annually. This study included 1,291 women for analysis, representing 20,579,272 women after weight calculation. Some limitations should be mentioned in this study. First, due to the cross-sectional design, the causation between PAH metabolites and endometriosis could not be determined, and the relationship between PAH exposure time and endometriosis could not be estimated. Second, many samples without urinary PAH examinations were excluded (*n* = 2,955), which may cause selection bias. However, PAHs were detected in 1/3 of the respondents from the population aged 6 and older surveyed by the NHANES, and we considered the corresponding weight in the analysis, so the bias is relatively small. Third, this study focused on the American population, the findings of which may have limited generalization. More investigations are warranted to certify these findings. Additionally, external data validation was not performed since the limited sample size. Future large-sample datasets are required for external validation. Because information on occupations was not available in the NHANES, which occupations have exposure to PAHs is unknown. Future studies could investigate this point.

## Conclusion

No significant association was found between individual PAH metabolites and the risk of endometriosis. A positive tendency existed between mixed PAH metabolites and endometriosis when all the metabolites were at their 60th percentile levels or above compared with their median levels, and when all the other metabolites were fixed at their median levels, 1-hydroxynaphthalene was positively correlated with endometriosis.

## Data availability statement

Publicly available datasets were analyzed in this study. This data can be found here: NHANES database, https://www.cdc.gov/nchs/nhanes/index.htm.

## Ethics statement

The requirement of ethical approval was waived by The First Affiliated Hospital of Soochow University for the studies involving humans because the data was accessed from a publicly available database. The studies were conducted in accordance with the local legislation and institutional requirements. The ethics committee/institutional review board also waived the requirement of written informed consent for participation from the participants or the participants’ legal guardians/next of kin because retrospective nature of the study.

## Author contributions

LZ: Data curation, Formal analysis, Funding acquisition, Investigation, Methodology, Project administration, Software, Supervision, Validation, Visualization, Writing – original draft, Writing – review & editing. XY: Data curation, Formal analysis, Investigation, Methodology, Project administration, Software, Supervision, Validation, Writing – review & editing.
