# Supplementary material for: Association between exposure to polycyclic aromatic hydrocarbons and endometriosis: data from the NHANES 2001–2006
Source: Front Public Health. 2024 Jan 8;11:1267124. doi: 10.3389/fpubh.2023.1267124 (PMC10801278; doi:10.3389/fpubh.2023.1267124)
Supplement: Supplementary file 1 [file Table_1.DOCX]

Supplementary Table 1 Study questions for variable assessment

| Variables | Study questions |
| --- | --- |
| Age at menarche | How old {were you/was SP} when {you/SP} had {your/her} first menstrual period? |
| Menopause | What is the reason that {you have/SP has} not had a period in the past 12 months? |
| Hysterectomy | {Have you/Has SP} had a hysterectomy that is, surgery to remove {your/her} uterus or womb? |
| Ovary removed | {Have you/Has SP} had at least one of {your/her} ovaries removed (either when {you/she} had {your/her} uterus removed or at another time)? |
| Female hormone use | {Have you/Has SP} ever used female hormones such as estrogen and progesterone? |
| Gravidity | {Do you/Does SP} think {you are/he/she is} pregnant now?/{Are you/Is SP} pregnant now? |
| Pregnancy times | How many times {have you/has SP} been pregnant? ({Again, be/Be} sure to count all {your/her} pregnancies including (current pregnancy,) live births, miscarriages, stillbirths, tubal pregnancies or abortions.) |
