# Supplementary material for: Association between exposure to polycyclic aromatic hydrocarbons and endometriosis: data from the NHANES 2001–2006
Source: Front Public Health. 2024 Jan 8;11:1267124. doi: 10.3389/fpubh.2023.1267124 (PMC10801278; doi:10.3389/fpubh.2023.1267124)
Supplement: Supplementary file 2 [file Table_2.DOCX]

Supplementary Table 2 Sensitivity analysis for the data before and after imputation

| Variables | Total (n=2582) | After imputation (n=1291) | Before imputation (n=1291) | Statistics | *P* |
| --- | --- | --- | --- | --- | --- |
| Education, n (%) |  |  |  | χ^2^=0.001 | 1.000 |
| College graduate or above | 582 (22.55) | 291 (22.54) | 291 (22.56) |  |  |
| Some college or AA degree | 856 (33.17) | 428 (33.15) | 428 (33.18) |  |  |
| High school graduate /GED | 575 (22.28) | 288 (22.31) | 287 (22.25) |  |  |
| Less than high school | 568 (22.01) | 284 (22.00) | 284 (22.02) |  |  |
| PIR, M (Q_1_, Q_3_) | 2.50 (1.20, 4.45) | 2.49 (1.21, 4.42) | 2.53 (1.17, 4.53) | Z=0.088 | 0.930 |
| Smoking, n (%) |  |  |  | χ^2^=0.000 | 0.988 |
| No | 1595 (61.80) | 798 (61.81) | 797 (61.78) |  |  |
| Yes | 986 (38.20) | 493 (38.19) | 493 (38.22) |  |  |
| Age at menarche, years, Mean ± SD | 12.61 ± 1.78 | 12.61 ± 1.78 | 12.61 ± 1.79 | t=0.03 | 0.975 |
| Pregnancy times, M (Q_1_, Q_3_) | 3.00 (2.00, 4.00) | 2.89 (2.00, 4.00) | 3.00 (2.00, 4.00) | Z=1.394 | 0.163 |
| BMI, kg/m^2^, Mean ± SD | 28.73 ± 7.25 | 28.73 ± 7.26 | 28.72 ± 7.25 | t=0.05 | 0.959 |
| Circumference, cm, Mean ± SD | 94.67 ± 16.07 | 94.76 ± 16.15 | 94.58 ± 16.00 | t=0.28 | 0.778 |
| Cotinine, ng/mL, M (Q_1_, Q_3_) | 0.07 (0.02, 3.33) | 0.07 (0.02, 4.29) | 0.06 (0.02, 2.79) | Z=-0.916 | 0.359 |

PIR, poverty-to-income ratio; BMI, body mass index; AA, associate; GED, general education development; SD, standard deviation; M, median; Q_1_, 1st quartile; Q_3_, 3st quartile.
